# Ancient mtDNA from the extinct Indian cheetah supports unexpectedly deep divergence from African cheetahs

**DOI:** 10.1038/s41598-020-60751-7

**Published:** 2020-03-12

**Authors:** Niraj Rai, Sunil Kumar Verma, Ajay Gaur, Florin Mircea Iliescu, Mukesh Thakur, Tirupathi Rao Golla, Kailash Chandra, Satya Prakash, Wajeeda Tabasum, Sreenivas Ara, Lalji Singh, Kumarasamy Thangaraj, Guy S. Jacobs

**Affiliations:** 1 Birbal Sahni Institute of Palaeosciences, Lucknow, India; 2 CSIR Centre for Cellular and Molecular Biology, Hyderabad, Telangana India; 3 Department of Genetics, University of Cambridge, Cambridge, UK; 4 Zoological Survey of India, Kolkata, India; 5 Ecological Genomics & Wildlife Conservation, Department of Zoology, University of Johannesburg, Johannesburg, South Africa; 6 Complexity Institute, Nanyang Technological University, Singapore, Singapore; 7 Department of Archaeology, University of Cambridge, Cambridge, UK

**Keywords:** Ecological genetics, Phylogenetics, Population genetics

## Abstract

The Indian cheetah was hunted to extinction by the mid-20th century. While analysis of 139 bp of mitochondrial DNA (mtDNA) has confirmed that the Indian cheetah was part of the Asiatic subspecies (*Acinonyx jubatus venaticus*), the detailed relationships between cheetah populations remains unclear due to limited genetic data. We clarify these relationships by studying larger fragments of cheetah mtDNA, both from an Indian cheetah museum specimen and two African cheetah, one modern and one historic, imported into India at different times. Our results suggest that the most recent common ancestor of cheetah mtDNA is approximately twice as ancient as currently recognised. The Indian and Southeast African (*Acinonyx jubatus jubatus*) cheetah mtDNA diverged approximately 72 kya, while the Southeast and Northeast African (*Acinonyx jubatus soemmeringii)* cheetah mtDNA diverged around 139 kya. Additionally, the historic African cheetah sampled from India proved to have an *A. j. jubatus* haplotype, suggesting a hitherto unrecognised South African route of cheetah importation into India in the 19^th^ century. Together, our results provide a deeper understanding of the relationships between cheetah subspecies, and have important implications for the conservation of *A. j. venaticus* and potential reintroduction of cheetahs into India.

## Introduction

In 1947, the last three confirmed Indian cheetahs (Asiatic cheetah subspecies, *Acinonyx jubatus venaticus*) were shot by the Maharaja of the State of Korwai^1^. Although the cheetah likely survived in isolated pockets for some time afterwards, this event was the final act in a long history of destructive human intervention on the subcontinent. Cheetah were caught in large numbers for coursing during the Mughal era^2^. The Mughal emperor Akbar (1556–1605) is said to have owned 9000 cheetahs over his lifetime. His coalition peaked at 1000 animals, and with only a single record of captive breeding nearly all of these were wild caught^3^. Anthropogenic pressure on Indian big cat populations continued during the British Raj, with estimates of over 80,000 tigers killed, for example^4^. Reports of cheetah hunting from this period are relatively rare, in part because the cheetah was already scarce^5^. The last confirmed population of the Asiatic cheetah (*Acinonyx jubatus venaticus*), the subspecies to which the Indian cheetah belonged, now live in Iran, and are themselves in a precarious conservation situation^6^.

Recently, there has been significant progress in understanding the genomic relationships between African cheetah subspecies^7^. This provides important phylogeographic context about the evolutionary history of cheetahs and can inform conservation decisions. Whole genome data from two closely related African subspecies, *Acinonyx jubatus jubatus* and *Acinonyx jubatus raineyi*, indicates that their common ancestor underwent a population expansion around 131 kya followed by the diversification into the two subgroups 24 kya and recent regional isolation and bottlenecks from 12 kya. While the expansion 131 kya has been interpreted as evidence of dispersals of modern cheetah from a putative American source population^7^, genetic evidence suggests that the ‘American cheetah’ are more closely related to pumas, such that modern cheetah likely have an Old World origin^8^ and this early expansion may reflect dispersals within Eurasia or Africa. In the absence of comparable genomic data from other cheetah populations it remains unclear whether this model – of a common ancestor from approximately 24 kya followed by recent isolation and dramatic population decline – is accurate for all cheetah subspecies, including the endangered Asiatic cheetah.

The limited amount of data that does exist from the Asiatic cheetah - small mtDNA fragments from modern Iranian samples and historic museum specimens (including just 139 bp from an Indian cheetah), coupled with limited autosomal microsatellite data from Iranian samples - have led to arguments for a somewhat deeper divergence between African and Asiatic cheetah of about 32–67 ky. Perhaps surprisingly, the Asiatic cheetah may have a closer mitochondrial relationship with the Southeast than Northeast African cheetah^9^. Interpreting these results in the context of the genomic model suggests regional population differentiation as a series of events in the late Pleistocene, including potentially complex dispersals between Africa and Asia. However, analyses by other authors^10^ using a larger mtDNA fragment does not replicate the closer association between Asiatic cheetah and Southeast African cheetah, and argues for a more recent divergence at 4.5–6.5 ky^10^, post-dating the start of the ongoing bottleneck among African cheetah over the last 12 ky^7^. We can therefore highlight two phylogeographic debates – the origin of modern cheetahs, likely resolved as Old World^8^, and the time scale of regional isolation, including differentiation between African and Asiatic cheetahs^7,9,10^. The latter question is particularly important given current discussion on the potential of re-establishing wild cheetah populations in India through the import of, most realistically, African animals^11,12^.

In this study, we seek to resolve the relationship between cheetah subspecies and address discrepancies between more ancient^9^ and more recent^10^ Asiatic and African cheetah divergence dates, testing the extent to which previous dating has been hampered by the limited genetic data available. To this end, we report and analyse mtDNA data recovered from a new historical Indian *A. j. venaticus* sample (Ind1), consisting of a much larger 4116 bp fragment of the mitochondrial genome. We additionally include two further samples to allow for more accurate dating, both imported to India at different times and of African ancestry (Ind2 and Ind3, see *Methods*). One of these provides the first full mtDNA of a Northeast African cheetah (*A. j. soemmeringii*) while the second is a partial mtDNA of an additional historical Southeast African cheetah sample. By combining these data sources we are able to finely date the matrilineal relationship and divergence of African and Asiatic subspecies. Our results emphasise the uniqueness of Asiatic cheetah, and the importance of conservation efforts.

## Results

### Sequencing of historical samples using complementary approaches yields rich and consistent data

Our sequencing experiments involved multiple complementary approaches (IonTorrent amplicon sequencing of the partial and full mitogenome, Sanger sequencing of PCR products, cloning experiments) to retrieve a substantial partial or full mtDNA data from the various samples and validate SNPs (see *Methods*).

For Ind1 the IonTorrent amplicon sequencing alignment consisted of 301,058 reads covering 6043 bp (of 17047 sites) with an average depth of 2042x. Our final called sequence covered 4116 bp with read depth 10x or above, containing 11 confidently called SNPs against the cheetah mtDNA reference (NC_005211). We were able to use the follow-up cloning experiment data to confirm 1854 bp, a region that included 7 of the 11 SNPs identified. Two SNPs (16474 G > A and 16488 C > T) in the control region were called as heterozygous from the NGS data but were accepted because i) they were validated by cloning, ii) sequence data from *A. j. venaticus* was available for one of the SNPs and confirmed it as previously observed in Asiatic cheetah and iii) the two SNPs were from the same reads.

For Ind2 the IonTorrent amplicon sequencing alignment consisted of 198,468 reads covering 7570 bp with an average depth of 1251x. We were additionally able to perform Sanger sequencing of 3885 bp of the mitogenome, which covered, and validated, one SNP identified in the NGS data (16456:C > T) and extended the total sequence covered. Our final Ind2 sequence covered 8327 bp containing 4 confidently called SNPs against the cheetah mtDNA reference.

The modern mitogenome (Ind3) long-range PCR NGS alignment covering the full mtDNA consisted of 297,892 reads with an average depth of 2692x. The amplicon NGS alignment used to validate SNPs consisted of 230,249 reads covering 9519 bp with an average depth of 1937x. Our final Ind3 sequence covered the full 17047 bp of the cheetah mtDNA reference and contained 89 SNPs.

### The three new cheetah mtDNA sequences cluster with known African and Asiatic cheetah subspecies

**Figure 1:**
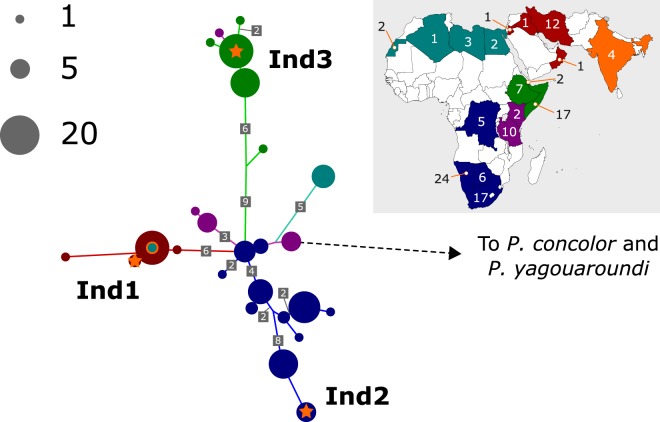
Tree schematic situating the Indian cheetah mtDNA within a worldwide sample of 121 modern and historic cheetah, based on a maximum likelihood unrooted tree using a concatenated alignment of up to 1238 bp. Colours indicate sampling location, and the numbers of samples from different countries is indicated on the map: green, northeast Africa; teal, northwest Africa; purple, east Africa; blue, southern Africa; dark red, southwest Asian; orange, Indian. These geographic sampling locations correlate strongly with mtDNA subspecies affiliation, though some samples are imports or customs seizures such that ancestral origin is ambiguous. The mtDNA samples presented in this paper (Ind1, Ind2 and Ind3) are indicated on the tree as orange stars. The most probable ancestral state at internal nodes was inferred by maximum likelihood and branches that represent >1 mutation are labelled with the number of mutations. Note that branch length does not always correspond closely with the inferred number of mutations due to varying probability of different mutations according to the mutation model.

We first confirmed that our newly generated sequences did not show genetic signals consistent with numts (nuclear mitochondrial DNA, caused by the transposition of mitochondrial DNA into the nuclear genome, which can confound mtDNA phylogenetic analysis; see *Methods* and Fig. S1). We then sought to situate the three mtDNA sequences within broader patterns of cheetah diversity. We conducted a phylogenetic analysis of 118 published cheetah mtDNA sequences from various regions (Table S1), along with our newly generated sequences (Fig. 1). We found strong agreement with the analysis of Charruau *et al*.^9^, with cheetah mtDNA segregating strongly based on sampling location. The three newly generated samples show affinity with three different cheetah populations, Asiatic (Ind1), Southeast African (Ind2) and Northeast African (Ind3). These different associations reflect recent translocation of the animals. While Ind1 is a museum specimen of a local cheetah from within India, the modern sample Ind3 was a recent gift to Hyderabad Zoo of Northeast African maternal descent. Ind2 is a museum specimen from Mysore, and clusters strongly with Southeast African cheetahs (*A. j. jubatus*).

### Analysis of the mtDNA coding region indicates deeper divergence between cheetah subspecies than previously reported

**Figure 2:**
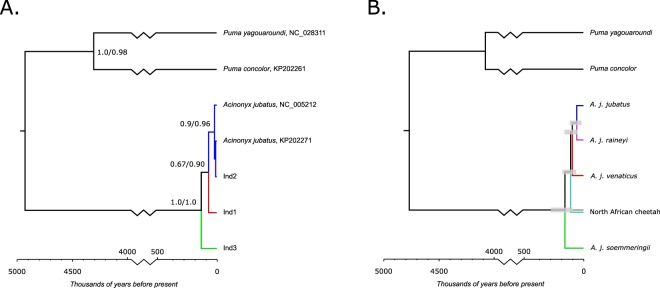
(**A**) Maximum likelihood phylogenetic tree generated using the mtDNA coding region, with puma and jaguarundi as an outgroup. Ind1, Ind2 and Ind3 represent newly reported samples. These three samples show considerable divergence, reflecting their mtDNA affinity with three different cheetah subspecies (see Fig. 1). Node annotation indicates branch support, based on SH-like approximately likelihood ratio (left) and BEAST posterior support for monophyly of taxa below nodes (right). (**B**) Maximum credibility BEAST reconstruction of cheetah subspecies relationships based on published short cheetah mtDNA fragments and the newly reported data. Ninety-five percent  highest posterior density intervals of population split times are shown. Branch colours correspond to cheetah subspecies: green, *A. j. soemmeringii*; teal, North Africa; purple, *A. j. raineyi*; blue, *A. j. jubatus*; dark red, *A. j. venaticus*.

We first analysed the mtDNA coding region of our newly reported data in the context of the small number of available full coding comparative sequences, using a maximum likelihood approach to build phylogenetic trees with puma (*Puma concolor;* KP202261) and jaguarundi (*Puma (Herpailurus) yagouaroundi;* NC_028311) as the outgroup. The best-supported tree topology is shown in Fig. 2A. This replicates expected mtDNA relationships^9^ in which the Asiatic cheetah and Southeast African are more closely related to one another than to Northeast African cheetah. Table S3 provides the relative support for the 10 most probable tree topologies. The five most probable topologies all support the placement of the Indian cheetah as outgrouping Southeast African cheetah mtDNA variation, with strong overall support (82%). There is low support (11.1%) for Ind1 mtDNA being situated within the Southeast African clade. Examination of branch lengths inferred for different tree topologies also indicates some support for models in which Ind1, Northeast African cheetah and the Southeast African cheetah clade represent a trifurcation (Table S3).

**Figure 3:**
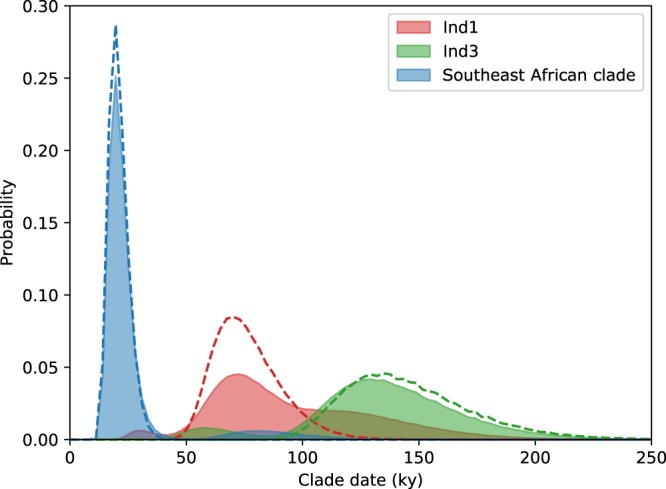
Clade date estimation based on maximum likelihood analysis of mtDNA (sequences as in Fig. 2). The dotted lines indicate the dates based on the most probable tree topology. The shaded regions show the effect of taking uncertainty in the tree topology into account by proportionally weighting the dates suggested by the 10 most-probable tree topologies (capturing 93% of probability according to the IQTree bp-RELL method).

The estimated mtDNA divergence dates between the Indian cheetah (Ind1) and both the Northeast African (Ind3) and Southeast African cheetah (NC_005212) assuming a fixed puma-cheetah divergence date of 4.92 million years^13^ are given for the 10 most probable topologies in Table S3. We also estimated divergence dates taking into account uncertainty in the tree depth and puma-cheetah divergence (see *Methods*). The tree topology with highest support suggests that the Indian cheetah split from the Northeast Africa cheetah 138.9 ky (100–205.6 ky) ago. This tree infers a split date of Indian cheetah mtDNA with Southeast African cheetah of 72.2 ky (50–108.3 ky). Additionally, taking into account uncertainty in tree topology (Fig. 3) by weighting the divergence date distributions from each tree by its relative probability yields divergence dates of 133.3 ky (52.8–200 ky) and 86.1 ky (33.3–172.2 ky), respectively.

We confirmed the accuracy of these dates by repeating the analysis in a Bayesian statistical framework, using BEAST^14^. This yielded consistent results, with inferred split times between the Indian cheetah sequence and the Northeast African cheetah sequence of 171.7 ky (95% HPD 103.6–244.9 ky) and between the Indian and Southeast African cheetahs 101.7 ky (95% HPD 43.8–177.8 ky). Both BEAST dates substantially overlap with their corresponding maximum likelihood dates. As before, the vast majority (90.3%) of sampled trees supported monophyly of the Indian cheetah (Ind1) and Southeast African mitochondrial DNA, to the exclusion of the Northeast African cheetah sequence.

To better assess mtDNA evidence for the divergence dates of cheetah subspecies, we extended the BEAST analysis to include the shorter mtDNA sequence fragments already visualised in Fig. 1 and reported in Table S1. This yielded a split between the Asiatic and Northeast African cheetah subspecies of 143.3 ky (95% HPD 75.1–217.2 ky) and between the Asiatic and Southeast African cheetah subspecies of 103 ky (95% HPD 45–173.1 ky). The maximum credibility subspecies tree is shown in Fig. 2B, and the maximum credibility sequence tree is shown in Fig. S2.

All analyses supported the closer mitochondrial association of Indian cheetah with the Southeast African over Northeast African subspecies, and reveal more ancient common ancestry dates than those proposed by Charruau *et al*.^9^ and O’Brien *et al*.^10^. To check whether any specific sequence was driving the high date estimate we repeated the maximum likelihood analysis i) excluding the Jaguarundi, ii) excluding the Ind3 Northeast Africa mtDNA sample and iii) excluding all samples except for Puma, Ind1 and the Southeast Africa cheetah mtDNA reference sequence. Based on the best supported tree topology in each case and assuming the fixed puma-cheetah divergence date of 4.92 million years, the divergence dates estimated based on these reanalyses were 76.6 ky (standard error 24.6 ky), 70.6 ky (24.6), and 64.6 ky (26.5) respectively. Repeating the analysis using the portion of the coding region sequenced in both the historical samples Ind1 and Ind2 (such that there was no missing data) yielded a divergence date between Indian and Southeast Africa cheetah of 71.4 ky (39).

Comparing the 3675 bp of Ind1 mtDNA coding sequence with the *A. j. jubatus* reference mtDNA (NC_005212) directly revealed variants at 6 sites, with a further 3 ambiguous sites. Removing the 3 ambiguous sites did not alter the date estimates, confirming that they are not driving the deeper divergence dates. The additional full *A. j. jubatus* mtDNA included in the analysis (KP202271) differed from the Indian cheetah coding region at 8 sites, suggesting that it is important to sample variation within the *A. j. jubatus* clade to obtain accurate divergence dates.

Together, our results support an unexpectedly deep mtDNA divergence between the Indian and Southeast and Northeast African cheetahs. They also clearly show a closer mitochondrial association between Indian and Southeast African than Northeast African cheetahs. We confirm that the shorter mtDNA sequences previously available were insufficient to finely resolve the divergence dates of cheetah subspecies.

## Discussion

The recent history of the cheetah in India is an unfortunate one. Its extinction, likely in the 1960s^3^, marked the end of a long period of decline driven by human action and intervention. The increasing conversion of grasslands for agriculture put significant habitat pressure on populations, which were further depleted due to the huge popularity of coursing in Indian courts from the 16th century especially^3^. The popularity of the sport waned as cheetah became increasingly rare in the 18th and 19th centuries, with trophy hunting during the British era and bounties on cheetah-killing further contributing to their decline^15^. At least 127 cheetahs are known to have been captured, killed or photographed between 1800–1950^3^ with records attesting to a minimum of 70 further cheetahs being killed for rewards offered by the colonial administration between 1870–1925^15^. Such was the prestige of coursing and the rarity of cheetahs that during the early 20th century a number – perhaps around 200, though it is hard to estimate from available sources – were imported from Africa as substitutes^3^.

By sequencing historical specimens, we are able to observe two stages of this history. Firstly, we genetically profile and confirm an Indian cheetah (Ind1) dating to early 19th century Madhya Pradesh. Secondly, we identify a cheetah that was potentially one of these 200 imported cheetah, kept in the Mysore Natural History Museum (Ind2). Intriguingly, this specimen shows mtDNA affinity with Southeast African cheetah. Divyabhanusinh, a major authority on the history of the Indian cheetah, quotes sources supporting the import of cheetah from East Africa especially [3, pp 153–154]. As such, the affiliation of our specimen with Southeast Africa (Fig. 1) may be surprising, and potentially indicates southern Africa as an additional historical source of African cheetah imported into India. It is not known whether this specific cheetah was used in coursing or brought for some other purpose. The use of museum specimens to study the genetics of extinct species and populations, or indeed to look beyond recent changes in the population size or geographic range of extant species, is a powerful and increasingly important molecular approach.

The maximum likelihood tree based on a large fragment of the mtDNA coding region (Fig. 2) yielded mtDNA split dates between the Northeast African cheetah and a clade including both Southeast African and Indian cheetah of 138.9 ky (100–205.6 ky), and between the Southeast African and Indian cheetah of 72.2 ky (50–108.3 ky). These divergence dates are important for two reasons. Firstly, they push back the mitochondrial relationship between African cheetah subspecies. Secondly, they suggest a deeper mtDNA split between Asiatic and Southeast African cheetah than is currently supposed^9,10^.

The divergence dates we report indicate that the Northeast African (*A. j. soemmeringii*) and Southeast African (*A. j. jubatus*) cheetah share their mitochondrial ancestor approximately 138.9 ky (100–205.6 ky) as compared to previous estimates of 66.5 ky (32.3–244 ky)^9^ and 4.9 ky^10^, Our deeper divergence date is substantially earlier than the time at which the East African (*A. j. raineyi*) and Southeast African (*A. j. jubatus*) populations separated based on genomic data (24 kya), and more closely aligns with the reported 131 kya population expansion^7^. While ancient mtDNA sequence coalescence is potentially consistent with recent subspecies divergence given the inferred large ancestral cheetah effective population size reported by Dobrynin *et al*. (N_e_ = 142,500^7^), estimates of subspecies divergence incorporating a larger dataset of small mtDNA fragments in a Bayesian framework also supported an ancient split between *A. j. jubatus* and *A. j. soemmeringii* of 143.3 ky (95% HPD 75.1–217.2 ky). The split between *A. j. jubatus* and *A. j. raineyi* was inferred by the BEAST analysis of the combined coding and short mtDNA fragment dataset as 85.8 ky (95% HPD 20.9–160.4 ky), which is generally greater than, but overlaps, the genomic date. The difference between mitochondrial and genomic dates emphasises the importance of ultimately including whole genome sequences from each cheetah subspecies for phylogeographic reconstruction in the future. Nevertheless, our results clearly argue against a very recent divergence of Southeast and Northeast African cheetah^10^.

The mtDNA split between Asiatic and Southeast African cheetah was reported by Charruau *et al*.^9^ as 41.9 ky (20.3–153.8 ky), based on a substantially shorter mtDNA fragment that the one analysed here. This date was determined using a substitution rate estimated from the observed genetic distance between cheetah and puma mtDNA calibrated to the 4.92 mya (95% CI = 3.86–6.92) puma-cheetah split date reported by Johnson *et al*.^13^. This is the same calibration date that we apply to the puma-cheetah split in our phylogenetic tree; though we are able to incorporate far longer mtDNA sequences and a more accurate representation of the puma clade outgroup by including a jaguarundi as well *P. concolor* in our analysis. While the mtDNA split between Asiatic and Southeast African cheetah reported by Charruau *et al*. is more recent than our estimate, the confidence intervals of the two dates substantially overlap^9^.

In contrast, O’Brien *et al*.^10^ estimate the divergence of Asiatic and various African cheetah mtDNA to be 4383–6576 years. This far more recent date is based on calibration of the mtDNA molecular clock to divergence of *A. j. jubatus* and *A. j. raineyi* estimated as 4.2–4.5 kya based on the rate at which microsatellite diversity recovers after a proposed ancestral bottleneck^16^. However, microsatellites show extreme mutation rate variation, such that accurate divergence date estimation is challenging without detailed information on the mutation rate of individual microsatellite markers. The proposed calibration date of 4.2–4.5 kya is in apparent disagreement with the *A. j. jubatus* and *A. j. raineyi* divergence date of 24 ky estimated using whole genome data^7^. Furthermore, the rapid mtDNA mutation rate implied by this calibration would suggest a vastly more recent puma-cheetah mtDNA split – re-calibrating our trees based on the suggested Namibia (*A. j. jubatus*) vs Somalia (*A. j. soemmeringii*) split date of 4877 years, for example, would lead to an inferred puma-cheetah mtDNA split of 170.8 ky (much more recent than e.g. the fossil calibration of >3.8 my applied by Johnson *et al*.^13^). The considerable lineage mutation rate heterogeneity required to incorporate both very recent cheetah diversification date and ancient puma-cheetah divergence is not supported in our analysis (indicated by the insignificant likelihood-ratio test for evidence of variable mutation rate, see *Methods*), and effects such as saturation of mutations are not likely in the coding region over these time scales. Thus, recent common mtDNA ancestry among cheetah subspecies dating to the last few thousand years is not supported.

We propose an alternative hypothesis. Charruau *et al*.^9^ studied microsatellite data from Iranian and African cheetahs, and estimated the most probable population divergence dates between Iranian *A. j. venaticus* and *A. j. jubatus* to between 4.7 and 67.4 kya, depending on the mutation rate and modelling approach. Considering the demographic model estimated by Dobrynin *et al*.^7^ using whole genome data, this period coincides with an era of relatively very large effective population sizes (N_e_) in the ancestors of African cheetah (prior to 12 kya, N_e_ ranges from 37,700 to 142,200). If the Asiatic cheetah diverged from African populations >12 kya then, in the context of the large population size of the common ancestor, their mtDNA is expected to coalesce at a considerably more ancient date. Even taking into account the smaller N_e_ expected for mtDNA as a uniparental haploid locus, our ancient mtDNA divergence dates are consistent with the population divergence dates suggested by Charruau *et al*.^9^ and large ancestral population sizes inferred by Dobrynin *et al*.^7^. Recent bottlenecks leading to few uncoalesced mitochondrial lineages from our sample prior to 50 kya (Fig. S2) complicate the estimation of ancestral population sizes and the reconstruction of subspecies split dates from mtDNA data. Nevertheless, our BEAST analysis incorporating a large number of short mtDNA fragments yielded *A. j. venaticus* and *A. j. jubatus* species split date of 103 ky (95% HPD 45–173.1 ky), which overlaps the microsatellite estimate^9^. Our results argue for more ancient cheetah mitochondrial divergence dates than have previously been reported, and deep structure among cheetah subspecies.

Interestingly, we confirm a closer mitochondrial association between the Asiatic cheetah and Southeast African, rather than Northeast African, cheetah^9^ (Fig. 2A). Indeed, a rapid sequence of branching events between the North African, Asiatic and Southeast African/East African clade is recorded in the BEAST analysis, dated to between 116.2 ky (95% HPD 59.5ky-174.6 ky) and 101.6 (95% HPD 52.1–160.1 ky) in the maximum credibility tree (Fig. 2B), and may reflect founder effects associated with a single geographic radiation, perhaps associated with climatic and ecological changes during the Late Pleistocene. Although genomic data supports early Late Pleistocene population growth^7^, the diversification of *A. j. jubatus* and *A. j. raineyi* was more recent, indicating that isolation between cheetah subspecies is unlikely to have been as complete and early as the mitochondrial data suggest and potentially supporting dates toward the lower limit of confidence bounds. We note that the length of mtDNA sequence available for most samples in this analysis is extremely limited, and that microsatellites tend to associate Southeast and Northeast African populations with Asiatic cheetah as the outgroup^9^. Further work is required to determine whether this signal is stable, demographically meaningful given the recent bottlenecks in cheetah populations, and consistent with autosomal data.

By incorporating substantially more historic sequence data and multiple modelling approaches, our study helps to resolve cheetah evolutionary history and has implications for current debates on conservation and the re-introduction of cheetah into India^10–12^. To summarise, our results argue for a history of divergence and local adaptation in Asiatic cheetah that is substantially more ancient than some recent date estimates^10^ and than the 12–24 ky timeframe for the split of East and Southeast African cheetahs^7^. The data is consistent with multiple bottlenecks affecting different cheetah subspecies, perhaps preceded by a radiation of a population that had already diverged from the ancestors of modern Northeast African cheetahs. It also supports substantial isolation of Asiatic cheetahs. The ancient divergence of mitochondrial haplogroups, their high frequency within groups, and the good representation of cheetah from both Africa (100 samples) and Asia (19 samples) in our dataset (Table S1) mean that substantial gene flow would be expected to leave a clear signal in haplotype distributions. However, only one out of the hundred samples originating in African in the dataset analysed (Table S1) showed an Asiatic haplogroup (from Egypt, in the 1850s^9^), and all Asian samples with African haplogroups are probable or known recent translocations. The lack of gene flow implied by mtDNA data is consistent with the greater *F*_*ST*_ between Asiatic and African populations reported based on autosomal microsatellite data^9^. While our account of divergence and isolation is based on the history of the single-locus, maternally inherited mtDNA, and while the combination of a large ancestral population size and extreme recent drift^7^, leading to slow coalescence of potentially divergent regional founder haplogroups, potentially complicates subspecies dating, we interpret our results as strongly supporting the genetic uniqueness of Asiatic cheetahs, and the importance of conservation efforts given their extreme rarity^6^.

This paper is a report on the genetic divergence of different cheetah subspecies, rather than a paper on the potential and pitfalls of cheetah re-introduction into India. Nevertheless, given – rightly or wrongly – the role of genetic studies in guiding this debate, some comment and reference to existing literature is appropriate. There are two fundamental questions – whether re-introductions should occur (if appropriate sites are identified^11^), and which subspecies is most appropriate if they do. In terms of the latter, it has repeatedly been noted that while Iranian and Indian cheetah are most closely related genetically, the Asiatic cheetah population is critically endangered in Iran and not sufficiently robust to sustain offtake for reintroduction^9,10,17^. It is furthermore probable that the level of genetic differentiation between African cheetah subspecies and Asiatic cheetah is not sufficiently great as to genetically impact the ability of African cheetah to survive in India^17^. Reassuringly, genetic modelling confirms that the severe population bottleneck of African cheetah is many thousands of years old^7^, suggesting that low diversity has not precluded the survival of cheetah over vast, and ecologically variable, regions in Africa over that period. Genetic studies, including our own, do not rule out re-introduction on genetic grounds. As has previously been argued, any choice between African subspecies would need to take similarity of ecology and behaviour into account^9^.

The question of whether re-introductions should occur is complex, and beyond the scope of this work. Experienced geneticists strongly support the reintroduction^10^, suitable sites have been discussed^11^, and the overall divergence between cheetah subspecies is not considered too large to preclude re-introduction^10,17^. Nevertheless, concerns remain^12^. While we see no genetic reason precluding re-introduction, consideration of local environments and ecology, animal behaviour, anthropogenic pressures, the likelihood of success, and the probable impact on other potentially endangered species is of course important.

In conclusion, by sequencing a substantially longer section of mtDNA in several historic and modern cheetah samples we have been able to refine the maternal picture of cheetah subspecies divergence. Our results suggest more ancient split dates than previously reported^9,10^, and hence emphasise the importance of cheetah conservation at the subspecies scale.

## Methods

### Sample collection

We collected samples from three cheetah located in India, two historic and one modern. The first historic sample, here identified as Ind1, came from the mammal gallery of the Zoological Survey of India (ZSI), Kolkata (Reg. No# 7611). This individual is believed to have been shot in Madhya Pradesh in the early 19th Century, and the skin was purchased from R. Ward & Co. and added to the gallery of the Indian Museum in Kolkata on 14.11.1996. Subsequently, this specimen was moved to the mammal gallery of ZSI, Kolkata. The second historic cheetah, here identified as Ind2, was a bone sample contributed by the Mysore Natural History Museum, and dates to 1850–1900. The modern sample, Ind3, was derived from excess blood gathered as part of a routine clinical examination and contributed in 2017 by the Nehru Zoological Park, Hyderabad, to CSIR-CCMB (MOU ref. 1145/2016/A6).

### Genetic sequencing

Ancient DNA (aDNA) extractions were performed in specialist aDNA-only facilities (positive pressure; physical separation of gowning, DNA extraction and library preparation rooms; regular decontamination) at the Centre for Cellular and Molecular Biology, Hyderabad. All post-PCR experiments were carried out at the modern DNA labs which are geographically distant from the aDNA facilities. Ancient DNA extraction followed a standard protocol^18^, using extraction buffer consisting of EDTA and Proteinase K for the digestion of skin sample (slightly modified, such that 20 mg/ml of Proteinase K was used rather than 10 mg/ml). Overnight incubation and complete digestion with the extraction buffer was followed by binding DNA to silica beads and DNA purification using guanidium thiocynate. DNA quantification of the extract was carried out using a Qubit fluorometer with concentrations in the range 100–150 pg/ml. Multiplex PCR was carried out using 2 ml of the ancient DNA extracts in a final volume of 20 ml PCR cocktails.

DNA extraction from the modern cheetah sample was completed using QIAamp DNA Blood Mini Kit (Qiagen), following the manufacturer’s protocol, in a separated modern DNA facility.

All DNA extracts (modern and ancient DNA) were quantified using Qubit fluorometer (Invitrogen, USA). The amplification products of long-range PCR were visualized on 2% agarose gel electrophoresis). Concentration and fragment size of the long-range PCR products were interpreted using quantitative ladders.

Due to the limited amount of DNA available and the historic origin of two of the samples we used multiple sequencing approaches.

### Sequencing the two historic samples, Ind1 and Ind2

We first designed multiplex primers to amplify up to 8 kb of the *A. j. jubatus* reference mitochondrial genome (GenBank accession NC_005212), consisting of three multiplex reactions having 35, 34 and 26 amplicons targeting the coding and HVS regions of the mtDNA. After initial experiments, 41 primers were found to amplify consistently (average amplicon size 82 bp; see Supplementary Information Table S4), and were used in a single multiplex PCR reaction for each sample.

Multiplex PCRs were performed using Taq polymerase from the Sequenom MassARRAY system (San Diego, CA) kit which is designed for mass array-based genotyping. Amplification was carried out in 50 µl reactions following the manufacturer’s instructions, using 2 ng of DNA and 100 µM of each primer. Multiplex PCRs were performed on a GeneAmp 9700 thermal cycler (Applied Biosystems) with the following PCR conditions: initial denaturation at 94 °C for 15 min for hot start, followed by 45 cycles of denaturation at 94 °C for 20 sec, annealing at 56 °C for 30 sec, extension at 72 °C for 1 min, and a final extension at 72 °C for 3 min. After the multiplex PCR reaction, the products were treated with shrimp alkaline phosphatase (SAP, Amersham, Freiburg, Germany) to dephosphorylate left over dNTPs. The reaction conditions were: 37 °C for 20 minutes to remove remaining dNTPs, followed by 85 °C for 30 minutes to inactivate SAP and final incubation at 4 °C until the plate was removed from the thermal cycler. After SAP treatment, PCR products were cleaned using MinElute PCR purification kit (Qiagen).

We performed Next Generation Sequencing (NGS) using multiplex PCR products using the Ion Torrent Personal Genome Machine (PGM) platform (and confirmed SNPs using Sanger sequencing, see below). We sequenced the pooled amplicons directly, without fragmentation and blunt-ended conversion, using the Ion Plus Fragment Library Kit.

The DNA was ligated to Ion-compatible barcode adapters (Ion Xpress Barcode Adapters Kits), followed by nick-repair to complete the linkage between adapters and DNA inserts and purification. The adapter-ligated libraries were then size-selected for optimum read length (E-Gel SizeSelect). The DNA libraries were purified with freshly prepared 70% ethanol and each barcoded library was analysed using a Bioanalyzer (Agilent High Sensitivity DNA Kit) to assess individual barcoded library size distribution and determine the molar concentration in pmol/L of each barcoded library, followed by sequencing on the PGM platform.

To validate SNPs and extend sequence coverage we additionally performed Sanger sequencing, either directly on the multiplexed fragments (for the Ind2 sample, which yielded higher-quality NGS data) or after cloning (for Ind1). For Ind1, we cloned the multiplex PCR products using blunt-ended PCR cloning kits (GE Healthcare, USA), which contain pMOSBlue dephosphorylated blunt end vector. Transformation was carried out using DH5 competent cells, as per the manufacturer guidelines. Transformed cells containing insert PCR products from each multiplex PCR pool was screened using blue-white selection of colonies. We picked up approximately 1000 white colonies, isolated plasmid DNA using plasmid isolation kit (Qiagen), and then sequenced (Sanger sequencing) using universal M13 primers. The Sanger sequencing was done using BigDye terminator cycle sequencing kit V3 (Applied Biosystems, USA). After the sequencing reaction, fragments were precipitated with 5 M sodium acetate and ethanol, washed twice with 70% alcohol, dried, dissolved in Hi-Di formamide, and sequenced using an ABI 3730 Genetic Analyzer (Applied Biosystems, Foster City, USA). Sequences were edited and assembled using Sequence Analysis and AutoAssembler software (Applied Biosystems, Foster City, USA), respectively.

### Sequencing the modern cheetah sample, Ind3

To sequence Ind3, we first performed long-range PCR followed by NGS of the full mtDNA. As an additional check, we also preformed amplicon NGS as described above, allowing us to validate SNPs in the sequenced region.

We collected 2 ml of blood from Ind3 and isolated DNA using a phenol-chloroform extraction. DNA was quantified using 0.8% agarose gel and Qubit 4 Fluorometer (Thermo Fisher Scientific, USA). 150 ng of DNA was used for the long-range PCR, using primers designed with Primer3Plus software^19^. Four pairs of oligos were used to amplify the complete mtDNA (primers and annealing temperatures are given in Table S2). QIAGEN Long Range PCR (cat. No. 206401) Kit was used to amplify the complete mtDNA genome, with 35-cycles of amplification yielding satisfactory results. PCR purification was done using QiAquick gel extraction kit, QIAGEN (Cat. No. 28704). We mixed the long-range PCR products in equimolar concentration, using a total of 1 mg of DNA for NGS library preparation. We fragmented the amplicons to 200 bp using Ion Shear Plus Enzyme Mix, and performed NGS sequencing on the Ion Torrent PGM platform as above. We obtained 600MB data with 2 million reads from the sequencing run.

### Bioinformatics and analyses

The three mitogenomes were reconstructed using next generation sequence (IonTorrent PGM), followed by validation of SNPs by various methods (see above). After exploring data quality using FastQC v0.11.5^20^, we used a custom script to drop unterminated reads and remove adaptors and primers. We then used Trimmomatic v0.34^21^ to trim low-quality reads (LEADING:20 TRAILING:20 SLIDINGWINDOW:4:15 MINLEN:18). We then aligned reads to the *A. j. jubatus* mtDNA reference sequence (NC_005212) using Bowtie 2.0^22^ and end-to-end alignment and the -very-sensitive flag. After converting to.bam format using samtools^23^ we proceeded with SNP calling using GATK v3.7-0gcfedb67^24^. We applied base recalibration using a manually generated set of reference SNPs based on published cheetah mtDNA (for a list of samples used see Table S1) before SNP calling using default parameter settings. We hard filtered SNP calls (DP < 10 || QD < 5.0 || FS > 10.0 || MQ < 20.0 || ReadPosRankSum <−8.0). A small number of SNPs were found to be heterozygous calls; these potentially reflect heteroplasmy, DNA damage or limited incorporation of numt sequence (common in Felidae, e.g.^25^, which can lead to chimeric reconstructions of nuclear and mitochondrial sequence and confound phylogenetic analysis). These were excluded unless they could be confirmed via a cloning experiment (two cases, see below). Sequence with read depth <10 was recorded as missing data in the analysis. As the mtDNA is relatively short, we were able to manually check called SNPs against the aligned reads using Tablet v1.16.09.06^26^, confirming that all called SNPs had considerable support.

To confirm the absence of numts in our sequence data, we followed Li *et al*.^27^ in plotting the rate of mismatch of newly generated sequences against high-quality published mtDNA data (here, the cheetah mtDNA reference sequence NC_005212, confirmed by Li *et al*. to be free from numts). We additionally plotted the mismatch rate of the *Puma concolor* mtDNA reference sequence NC_016470, thought to incorporate a numt spanning the 12 S and 16 S genes^27^, against a high quality *Puma concolor* sequence (KP202261) as an example of the pattern expected when numts are incorporated into mtDNA data. This analysis, shown in Fig. S1, was conducted using a sliding 1000 bp window (values are not shown if <200 bp of the window was called), and confirms that our approach to SNP calling was effective in avoiding numts.

To locate the Indian cheetah mtDNA in the context of worldwide diversity we built a comparative dataset using published cheetah mtDNA data^9,27–29^ along with puma (*Puma concolor*) and jaguarundi (*Puma (Herpailurus) yagouaroundi*) mtDNA to provide an outgroup clade (see Table S1). This dataset yielded an alignment derived from both the mtDNA coding (ND5 and Cytochrome B) and control regions, concatenating to a length of 1238 bp; sequences from different data sources had different gaps in this alignment. It was possible to use the *Puma concolor* mtDNA reference sequence NC_016470 for this analysis as numts were not incorporated into these genes^27^. The dataset was aligned using a MAAFT online server^30^ and a consensus maximum likelihood phylogenetic tree was generated using IQTree online server model selection (supporting a TN + I model by Bayesian Information Criterion) and tree building methods^31^. We inferred probable mutations on the tree using the IQTree ancestral sequence reconstruction option (-asr) to infer the most likely sequence at each internal node. The location of the three Indian samples on this unrooted tree is indicated in the Fig. 1 schematic, where highly similar samples have been collapsed to aid visualisation.

To determine divergence dates between the mtDNA of cheetah subspecies, we constructed a maximum likelihood tree based on the coding region of available cheetah full mtDNA sequences, our Indian samples, and an outgroup consisting of puma (*Puma concolor,* KP202261) and jaguarundi (*Puma (Herpailurus) yagouaroundi*, NC_028311) mtDNA. As before, the IQTree web server was used to build a tree topology, this time using the TN + G5 model (the most strongly supported commonly implemented substitution model by BIC). We used PAML 4.7^32^ to retrieve likelihoods for a likelihood-ratio test of the molecular clock, and were unable to reject the null hypothesis that the rate of evolution is homogenous among all branches in the phylogeny (no clock log-likelihood = −29341.106115; strict clock log-likelihood = −29345.873227; p-value with 5 degrees of freedom = 0.09). The output tree from the strict clock model is shown in Fig. 2. The tree was scaled following Charruau *et al*.^9^, using a divergence date between the puma lineage and cheetah of 4.92 million years^13^.

As there were only seven branches in our divergence-dating dataset we were able to explicitly compare and assign probabilities to all possible tree topologies. We sought to retrieve as accurate as possible an estimate of divergence dates and topology support. All possible topologies were generated using a custom Python script (www.github.com/guysjacobs/cheetahtree) and used as input into IQTree. We used the bootstrap proportion using resampling estimated log-likelihoods method (bp-RELL^33^), to assign posterior weights to each topology, and retrieved the top 10 topologies which together accounted for 93.2% of the posterior weight. We used PAML to retrieve branch lengths from each tree. We then calculated the mutation rate separately for each tree topology, this time using the root height (and root height standard error, assuming a normal distribution of root height uncertainty) and a calibration distribution of the puma-cheetah divergence specifically, a skew-normal distribution fitted to the 4.92 my date and 3.86–6.92 my confidence intervals given in Johnson *et al*.^13^ using scipy.stats^34^. We multiplied the pdfs of these distributions to obtain relative probabilities of combinations of root height and puma-cheetah divergence, calculated the mutation rate implied by each combination of root height and puma-cheetah divergence, and, by weighting mutation rate by probability, obtain a distribution of mutation rate that takes into account both tree height uncertainty and uncertainty in the puma-cheetah divergence date. The mutation rate distribution based on each tree topology was used to convert branch lengths into date distributions. By weighting the divergence dates implied by each tree according to that tree topology’s bp-RELL posterior weight we obtained distributions of dates that take into account uncertainty in tree depth, the puma-cheetah divergence calibration point, and tree topology (Fig. 3); and by comparing the topology of trees we can calculate overall support for different evolutionary relationships in our data.

As an additional check on the divergence dates inferred by maximum likelihood, we conducted a Bayesian phylogenetic analysis using BEAST v2.4.8^14^. We used the same coding region alignment as in the maximum likelihood analysis above. As our data incorporated multiple species (puma, jaguarundi, cheetah subspecies) we used the starbeast2 package^35^. The xml file is available as Supplementary File S1. We used the Tamura-Nei mutation model with 5 Gamma rate categories, with a strict clock model, a birth-death tree prior and constant population sizes. Priors were left as default, with the exception of the strictClockRate (LogNormal with mean in real space = 1.0 and standard deviation 3.0) and constPopMean (LogNormal with mean = 1.0 and standard deviation 3.0). Calibration was achieved by fixing a prior on the species tree root (LogNormal with mean = 0.82 and standard deviation 0.33, and offset 2.65) that closely approximates the puma-cheetah divergence date distribution (4.92 mya, 95% CI = 3.86–6.92) suggested by Johnson *et al*.^13^. The MCMC chain length was set to 1e8, sampling every 20000 steps, and all effective sample sizes were over 500. As further checks on our BEAST results we both sampled directly from the priors (leading to extremely different posterior parameter estimates and hence confirming that the data drives model inference) and confirmed that using alternative models did not substantially impact estimated divergence times (Yule tree prior and/or a linear with constant root population sizes). The BEAST analysis confirmed the maximum likelihood tree topology (Fig. 2A).

To more accurately estimate cheetah subspecies divergence we modified the BEAST analysis to include the full dataset of published shorter mtDNA fragments (Table S1). The mixed nature of this dataset (with single species represented by both modern and historical samples) meant that it was not possible to directly model the ages of samples in the analysis, and all samples were assumed by the model to represent modern individuals. However, this will not greatly distort results as the age of most samples (<200 years) is a small fraction of the inferred date of the demographic events of interest (tens of thousands of years). Two somewhat older samples were included in the analysis (800–900CE, Asiatic cheetahs from Iran), but excluding these from the alignment and re-running the analysis lead to minimal changes in results (e.g. <3% difference in subspecies most recent common ancestor date estimates for Asiatic versus either Southeast or Northeast African cheetahs).

As this alignment included the D-loop region we performed the starbeast2 analysis using two partitions (coding region and D-loop) but linked trees, thus maintaining phylogenetic consistency while allowing the inference to incorporate a faster D-loop mutation rate. We excluded a repetitive region of the D-loop from this analysis (from position 257 to 594 of the cheetah mitochondrial reference sequence, NC_005212). A ‘Linear with Constant Root Populations’ model was preferred to allow for constant rate population size changes occurring over the time frame of species existence; but date estimates are robust to using different population modes (‘Constant Populations’). As above, we used the Tamura-Nei mutation model with 5 Gamma rate categories, with a strict clock model and a birth-death tree prior. Priors were set to default distributions except for the mutation rate (LogNormal with mean in real space = 1.0 and standard deviation = 3.0) and puma-cheetah divergence calibration as above. The MCMC chain length was set to 1e8, sampling every 20000 steps, and all effective sample sizes were over 500, with the TreeAnnotator v2.4.0 BEAST sub-program used to construct the maximum clade credibility tree shown in Fig. 2B. The .xml file is available as Supplementary File S2.

The reconstructed Ind1, Ind2 and Ind3 sequences are deposited on GenBank with accession numbers MK469961-3. Ion Torrent PGM output for each sequence is available from ENA (Accession ERP113364).

## Supplementary information

Supplementary Information.
