# Supplementary material for: Ancient mtDNA from the extinct Indian cheetah supports unexpectedly deep divergence from African cheetahs
Source: Sci Rep. 2020 Mar 12;10:4618. doi: 10.1038/s41598-020-60751-7 (PMC7067882; doi:10.1038/s41598-020-60751-7)
Supplement: Supplementary file 1 — Supplementary Information. [file 41598_2020_60751_MOESM1_ESM.pdf]

Ancient mtDNA from the extinct Indian cheetah supports unexpectedly deep divergence from African cheetahs

Niraj Rai, Sunil Kumar Verma, Ajay Gaur, Florin Mircea Iliescu, Mukesh Thakur, Tirupathi Rao Golla, Kailash Chandra, Satya Prakash, Wajeeda Tabasum, Sreenivas Ara, Lalji Singh, Kumarasamy Thangaraj, Guy S. Jacobs

Supplementary Information

Supplementary Figure S1. Plot of the average mismatch against high confidence mtDNA sequences calculated in sliding windows of 1500bp. The Puma concolor sequence NC_016470 was suggested to be impacted by a numt by Li et al. (2016), and replicating their analysis by calculating the mismatch against a higher confidence puma mtDNA sequence (KP202261) confirms this assessment. Newly generated cheetah sequences were compared to the Acinonyx jubatus mtDNA reference sequence (NC_005212), which was confirmed to be numt-free by Li et al. Small peaks are visible in the sequences of historic samples Ind1 and Ind2 due to regions with high rates of missing data (no value is reported when less than 100 sites are called in a 1.5kb window). The highest mismatch peak among the cheetah sequences is about a quarter of the height of the NC_016470 numt peak, indicating that our sequencing and SNP calling protocol was effective at avoiding numts.

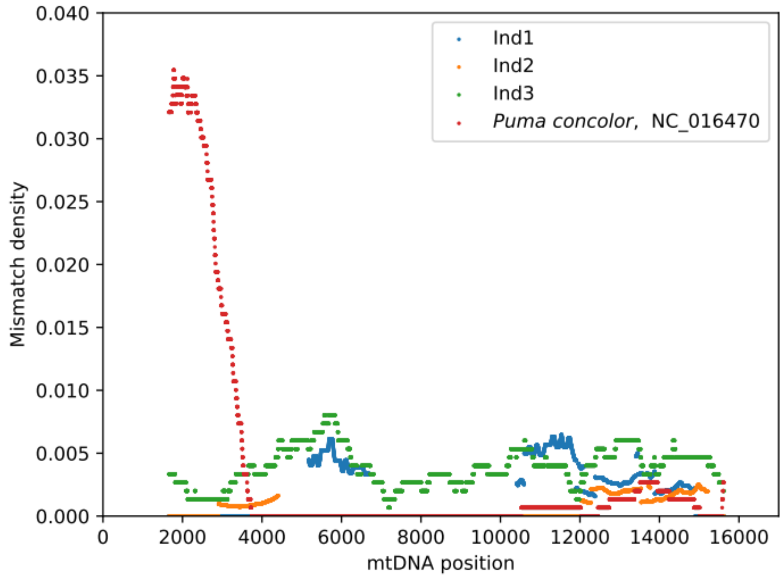

Supplementary Figure S2. BEAST phylogenetic tree of concatenated coding and D-loop mtDNA alignment, incorporating both full mtDNA sequences and fragments to infer subspecies divergence, see Methods and Supplementary File S2.

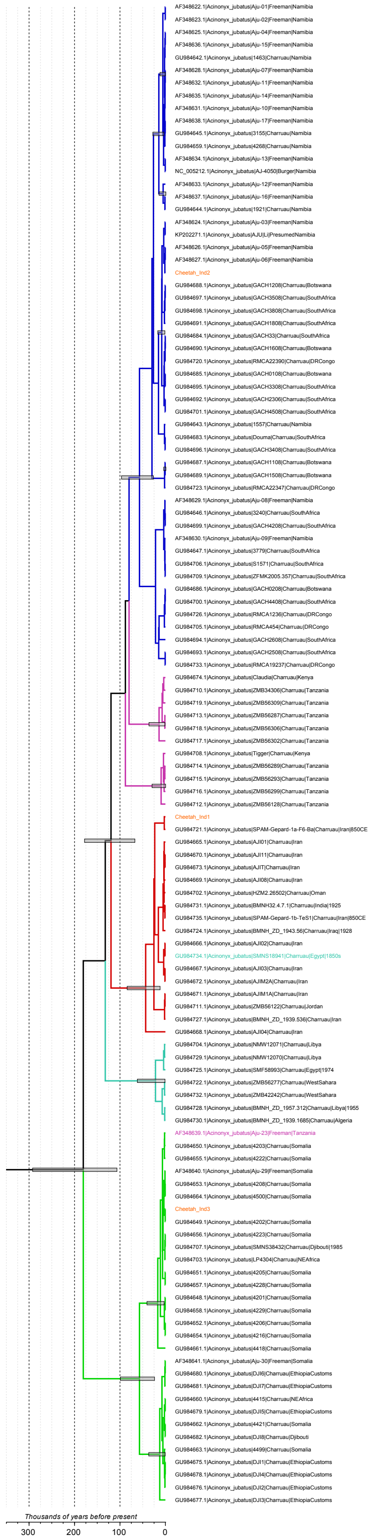

Supplementary Table S1. Samples used for SNP discovery (excluding MK469961-3) and exploring global diversity patterns (Main Text Figures 1 and 2B; SI Figure S2)

Genbank ID	Species	Sample ID	Study	Sample origin	Notes	mtDNA classification for BEAST analysis (red - probable migrant)
NC_028311	*Puma yagouaroundi*	HJA	Li *et al* 2016			*P. yagouaroundi*
NC_016470	*Puma concolor*	PC110509	Unpublished		Sequence incorporating numts not used. KP202261 used in other analyses.	*P. concolor*
NC_005212	*Acinonyx jubatus*	AJ-4050	Burger	Namibia		*A. j. jubatus*
KP202271	*Acinonyx jubatus*	AJU	Li *et al* 2016			*A. j. jubatus*
MK469963	*Acinonyx jubatus*	Ind1	*This study*	Madhya Pradesh, India	Early 19th century	*A. j. venaticus*
MK469962	*Acinonyx jubatus*	Ind2	*This study*	India	1850-1900	*A. j. jubatus*
MK469961	*Acinonyx jubatus*	Ind3	*This study*	India		*A. j. soemmeringii*
AF348641	*Acinonyx jubatus*	Aju-30	Freeman *et al* 2001	Somalia		*A. j. soemmeringii*
AF348640	*Acinonyx jubatus*	Aju-29	Freeman *et al* 2001	Somalia		*A. j. soemmeringii*
AF348639	*Acinonyx jubatus*	Aju-23	Freeman *et al* 2001	Tanzania		*A. j. soemmeringii*
AF348638	*Acinonyx jubatus*	Aju-17	Freeman *et al* 2001	Namibia		*A. j. jubatus*
AF348637	*Acinonyx jubatus*	Aju-16	Freeman *et al* 2001	Namibia		*A. j. jubatus*
AF348636	*Acinonyx jubatus*	Aju-15	Freeman *et al* 2001	Namibia		*A. j. jubatus*
AF348635	*Acinonyx jubatus*	Aju-14	Freeman *et al* 2001	Namibia		*A. j. jubatus*
AF348634	*Acinonyx jubatus*	Aju-13	Freeman *et al* 2001	Namibia		*A. j. jubatus*
AF348633	*Acinonyx jubatus*	Aju-12	Freeman *et al* 2001	Namibia		*A. j. jubatus*
AF348632	*Acinonyx jubatus*	Aju-11	Freeman *et al* 2001	Namibia		*A. j. jubatus*
AF348631	*Acinonyx jubatus*	Aju-10	Freeman *et al* 2001	Namibia		*A. j. jubatus*
AF348630	*Acinonyx jubatus*	Aju-09	Freeman *et al* 2001	Namibia		*A. j. jubatus*
AF348629	*Acinonyx jubatus*	Aju-08	Freeman *et al* 2001	Namibia		*A. j. jubatus*
AF348628	*Acinonyx jubatus*	Aju-07	Freeman *et al* 2001	Namibia		*A. j. jubatus*
AF348627	*Acinonyx jubatus*	Aju-06	Freeman *et al* 2001	Namibia		*A. j. jubatus*
AF348626	*Acinonyx jubatus*	Aju-05	Freeman *et al* 2001	Namibia		*A. j. jubatus*

AF348625	*Acinonyx jubatus*	Aju-04	Freeman *et al* 2001	Namibia		*A. j. jubatus*
AF348624	*Acinonyx jubatus*	Aju-03	Freeman *et al* 2001	Namibia		*A. j. jubatus*
AF348623	*Acinonyx jubatus*	Aju-02	Freeman *et al* 2001	Namibia		*A. j. jubatus*
AF348622	*Acinonyx jubatus*	Aju-01	Freeman *et al* 2001	Namibia		*A. j. jubatus*
GU984719	*Acinonyx jubatus*	ZMB56309	Charruau *et al* 2011	Tanzania		*A. j. raineyi*
GU984718	*Acinonyx jubatus*	ZMB56306	Charruau *et al* 2011	Tanzania		*A. j. raineyi*
GU984717	*Acinonyx jubatus*	ZMB56302	Charruau *et al* 2011	Tanzania		*A. j. raineyi*
GU984716	*Acinonyx jubatus*	ZMB56299	Charruau *et al* 2011	Tanzania		*A. j. raineyi*
GU984715	*Acinonyx jubatus*	ZMB56293	Charruau *et al* 2011	Tanzania		*A. j. raineyi*
GU984714	*Acinonyx jubatus*	ZMB56289	Charruau *et al* 2011	Tanzania		*A. j. raineyi*
GU984713	*Acinonyx jubatus*	ZMB56287	Charruau *et al* 2011	Tanzania		*A. j. raineyi*
GU984712	*Acinonyx jubatus*	ZMB56128	Charruau *et al* 2011	Tanzania		*A. j. raineyi*
GU984711	*Acinonyx jubatus*	ZMB56122	Charruau *et al* 2011	Jordan		*A. j. venaticus*
GU984710	*Acinonyx jubatus*	ZMB34306	Charruau *et al* 2011	Tanzania		*A. j. raineyi*
GU984709	*Acinonyx jubatus*	ZFMK2005.357	Charruau *et al* 2011	South Africa		*A. j. jubatus*
GU984708	*Acinonyx jubatus*	Tigger	Charruau *et al* 2011	Kenya		*A. j. raineyi*
GU984707	*Acinonyx jubatus*	SMNS38432	Charruau *et al* 2011	Djibouti	<1985	*A. j. soemmeringii*
GU984706	*Acinonyx jubatus*	S1571	Charruau *et al* 2011	South Africa		*A. j. jubatus*
GU984705	*Acinonyx jubatus*	RMCA454	Charruau *et al* 2011	DR Congo		*A. j. jubatus*
GU984704	*Acinonyx jubatus*	NMW12071	Charruau *et al* 2011	Libya		*North African*
GU984703	*Acinonyx jubatus*	LP4304	Charruau *et al* 2011	Northeast Africa		*A. j. soemmeringii*
GU984702	*Acinonyx jubatus*	HZM2.26502	Charruau *et al* 2011	Oman	1977	*A. j. venaticus*
GU984701	*Acinonyx jubatus*	GACH4508	Charruau *et al* 2011	South Africa		*A. j. jubatus*
GU984700	*Acinonyx jubatus*	GACH4408	Charruau *et al* 2011	South Africa		*A. j. jubatus*
GU984699	*Acinonyx jubatus*	GACH4208	Charruau *et al* 2011	South Africa		*A. j. jubatus*
GU984698	*Acinonyx jubatus*	GACH3808	Charruau *et al* 2011	South Africa		*A. j. jubatus*
GU984697	*Acinonyx jubatus*	GACH3508	Charruau *et al* 2011	South Africa		*A. j. jubatus*
GU984696	*Acinonyx jubatus*	GACH3408	Charruau *et al* 2011	South Africa		*A. j. jubatus*
GU984695	*Acinonyx jubatus*	GACH3308	Charruau *et al* 2011	South Africa		*A. j. jubatus*
GU984694	*Acinonyx jubatus*	GACH2608	Charruau *et al* 2011	South Africa		*A. j. jubatus*
GU984693	*Acinonyx jubatus*	GACH2508	Charruau *et al* 2011	South Africa		*A. j. jubatus*
GU984692	*Acinonyx jubatus*	GACH2306	Charruau *et al* 2011	South Africa		*A. j. jubatus*
GU984691	*Acinonyx jubatus*	GACH1808	Charruau *et al* 2011	South Africa		*A. j. jubatus*
GU984690	*Acinonyx jubatus*	GACH1608	Charruau *et al* 2011	Botswana		*A. j. jubatus*
GU984689	*Acinonyx jubatus*	GACH1508	Charruau *et al* 2011	Botswana		*A. j. jubatus*
GU984688	*Acinonyx jubatus*	GACH1208	Charruau *et al* 2011	Botswana		*A. j. jubatus*
GU984687	*Acinonyx jubatus*	GACH1108	Charruau *et al* 2011	Botswana		*A. j. jubatus*
GU984686	*Acinonyx jubatus*	GACH0208	Charruau *et al* 2011	Botswana		*A. j. jubatus*

GU984685	*Acinonyx jubatus*	GACH0108	Charruau *et al* 2011	Botswana		*A. j. jubatus*
GU984684	*Acinonyx jubatus*	GACH33	Charruau *et al* 2011	South Africa		*A. j. jubatus*
GU984683	*Acinonyx jubatus*	Douma	Charruau *et al* 2011	South Africa		*A. j. jubatus*
GU984682	*Acinonyx jubatus*	DJI8	Charruau *et al* 2011	Djibouti		*A. j. soemmeringii*
GU984681	*Acinonyx jubatus*	DJI7	Charruau *et al* 2011	Ethiopia Customs Seizure		*A. j. soemmeringii*
GU984680	*Acinonyx jubatus*	DJI6	Charruau *et al* 2011	Ethiopia Customs Seizure		*A. j. soemmeringii*
GU984679	*Acinonyx jubatus*	DJI5	Charruau *et al* 2011	Ethiopia Customs Seizure		*A. j. soemmeringii*
GU984678	*Acinonyx jubatus*	DJI4	Charruau *et al* 2011	Ethiopia Customs Seizure		*A. j. soemmeringii*
GU984677	*Acinonyx jubatus*	DJI3	Charruau *et al* 2011	Ethiopia Customs Seizure		*A. j. soemmeringii*
GU984676	*Acinonyx jubatus*	DJI2	Charruau *et al* 2011	Ethiopia Customs Seizure		*A. j. soemmeringii*
GU984675	*Acinonyx jubatus*	DJI1	Charruau *et al* 2011	Ethiopia Customs Seizure		*A. j. soemmeringii*
GU984674	*Acinonyx jubatus*	Claudia	Charruau *et al* 2011	Kenya		*A. j. raineyi*
GU984673	*Acinonyx jubatus*	AJIT	Charruau *et al* 2011	Iran		*A. j. venaticus*
GU984672	*Acinonyx jubatus*	AJIM2A	Charruau *et al* 2011	Iran		*A. j. venaticus*
GU984671	*Acinonyx jubatus*	AJIM1A	Charruau *et al* 2011	Iran		*A. j. venaticus*
GU984670	*Acinonyx jubatus*	AJI11	Charruau *et al* 2011	Iran		*A. j. venaticus*
GU984669	*Acinonyx jubatus*	AJI08	Charruau *et al* 2011	Iran		*A. j. venaticus*
GU984668	*Acinonyx jubatus*	AJI04	Charruau *et al* 2011	Iran		*A. j. venaticus*
GU984667	*Acinonyx jubatus*	AJI03	Charruau *et al* 2011	Iran		*A. j. venaticus*
GU984666	*Acinonyx jubatus*	AJI02	Charruau *et al* 2011	Iran		*A. j. venaticus*
GU984665	*Acinonyx jubatus*	AJI01	Charruau *et al* 2011	Iran		*A. j. venaticus*
GU984664	*Acinonyx jubatus*	4500	Charruau *et al* 2011	Somalia		*A. j. soemmeringii*
GU984663	*Acinonyx jubatus*	4499	Charruau *et al* 2011	Somalia		*A. j. soemmeringii*
GU984662	*Acinonyx jubatus*	4421	Charruau *et al* 2011	Somalia		*A. j. soemmeringii*
GU984661	*Acinonyx jubatus*	4418	Charruau *et al* 2011	Somalia		*A. j. soemmeringii*
GU984660	*Acinonyx jubatus*	4415	Charruau *et al* 2011	Northeast Africa		*A. j. soemmeringii*
GU984659	*Acinonyx jubatus*	4268	Charruau *et al* 2011	Namibia		*A. j. jubatus*

GU984658	*Acinonyx jubatus*	4229	Charruau *et al* 2011	Somalia		*A. j. soemmeringii*
GU984657	*Acinonyx jubatus*	4228	Charruau *et al* 2011	Somalia		*A. j. soemmeringii*
GU984656	*Acinonyx jubatus*	4223	Charruau *et al* 2011	Somalia		*A. j. soemmeringii*
GU984655	*Acinonyx jubatus*	4222	Charruau *et al* 2011	Somalia		*A. j. soemmeringii*
GU984654	*Acinonyx jubatus*	4216	Charruau *et al* 2011	Somalia		*A. j. soemmeringii*
GU984653	*Acinonyx jubatus*	4208	Charruau *et al* 2011	Somalia		*A. j. soemmeringii*
GU984652	*Acinonyx jubatus*	4206	Charruau *et al* 2011	Somalia		*A. j. soemmeringii*
GU984651	*Acinonyx jubatus*	4205	Charruau *et al* 2011	Somalia		*A. j. soemmeringii*
GU984650	*Acinonyx jubatus*	4203	Charruau *et al* 2011	Somalia		*A. j. soemmeringii*
GU984649	*Acinonyx jubatus*	4202	Charruau *et al* 2011	Somalia		*A. j. soemmeringii*
GU984648	*Acinonyx jubatus*	4201	Charruau *et al* 2011	Somalia		*A. j. soemmeringii*
GU984647	*Acinonyx jubatus*	3779	Charruau *et al* 2011	South Africa		*A. j. jubatus*
GU984646	*Acinonyx jubatus*	3240	Charruau *et al* 2011	South Africa		*A. j. jubatus*
GU984645	*Acinonyx jubatus*	3155	Charruau *et al* 2011	Namibia		*A. j. jubatus*
GU984644	*Acinonyx jubatus*	1921	Charruau *et al* 2011	Namibia		*A. j. jubatus*
GU984643	*Acinonyx jubatus*	1557	Charruau *et al* 2011	Namibia		*A. j. jubatus*
GU984642	*Acinonyx jubatus*	1463	Charruau *et al* 2011	Namibia		*A. j. jubatus*
GU984735	*Acinonyx jubatus*	SPAM-Gepard-1b-TeS1	Charruau *et al* 2011	Iran	800-900CE	*A. j. venaticus*
GU984734	*Acinonyx jubatus*	SMNS18941	Charruau *et al* 2011	Egypt	1850s	*A. j. venaticus*
GU984733	*Acinonyx jubatus*	RMCA19237	Charruau *et al* 2011	DR Congo		*A. j. jubatus*
GU984732	*Acinonyx jubatus*	ZMB42242	Charruau *et al* 2011	West Sahara		*North African*
GU984731	*Acinonyx jubatus*	BMNH32.4.7.1	Charruau *et al* 2011	India	1925	*A. j. venaticus*
GU984730	*Acinonyx jubatus*	BMNH_ZD_1939.1685	Charruau *et al* 2011	Algeria	1939	*North African*
GU984729	*Acinonyx jubatus*	NMW12070	Charruau *et al* 2011	Libya		*North African*
GU984728	*Acinonyx jubatus*	BMNH_ZD_1957.312	Charruau *et al* 2011	Libya	1955	*North African*
GU984727	*Acinonyx jubatus*	BMNH_ZD_1939.536	Charruau *et al* 2011	Iran		*A. j. venaticus*
GU984726	*Acinonyx jubatus*	RMCA1236	Charruau *et al* 2011	DR Congo		*A. j. jubatus*
GU984725	*Acinonyx jubatus*	SMF58993	Charruau *et al* 2011	Egypt	1974	*North African*

GU984724	*Acinonyx jubatus*	BMNH_ZD_1 943.56	Charruau *et al* 2011	Iraq	1928	*A. j. venaticus*
GU984723	*Acinonyx jubatus*	RMCA22347	Charruau *et al* 2011	DR Congo		*A. j. jubatus*
GU984722	*Acinonyx jubatus*	ZMB56277	Charruau *et al* 2011	West Sahara		*North African*
GU984721	*Acinonyx jubatus*	SPAM-Gepard-1a-F6-Ba	Charruau *et al* 2011	Iran	800-900CE	*A. j. venaticus*
GU984720	*Acinonyx jubatus*	RMCA22390	Charruau *et al* 2011	DR Congo		*A. j. jubatus*

Supplementary Table S2. Primers used for Long Range PCR.

	Forward Primer sequence (5'->3')	Reverse Primer (5'->3')	Amplicon Length	Nucleotide Positions	Tm (Forward Primer)	Tm (Reverse Primer)	Annealing Temp. °c	Concentration of PCR product in Nanogram
Primer Pair 01	ATACCCATACTGTGCTTGCCC	AGTACCGGCTCATAGTGGGATA	9839	146-9984	60.13	59.89	58	200 ng/ul
Primer Pair 02	ACCGACTGATTAATAACCGCCT	AGTCATAGCAAGTTCGACCCC	8195	9752- 17946	59.57	59.79	60	50ng/ul
Primer Pair 03	CCGACTGATTAATAACCGCCT	GTCATAGCAAGTTCGACCCC	8193	9753-17945	57.89	58.35	58	60ng/ul
Primer Pair 04	AACCGACTGATTAATAACCGCCT	GTGGAGACCCCGCATAGAG	8269	9751-18019	60.12	59.26	58	90ng/ul

Supplementary Table S3. Relative support (bp-RELL) of the 10 most supported tree topologies and divergence dates supported by each topology.

Tree topology	bp-RELL	Divergence (Indian and NE Africa, ky)		Divergence (Indian and SE Africa, ky)	
		ML	SD	ML	SD
((PC,PY),(I3,(I1,(AJ1,(I2,AJ2)))));	0.355	140.5	18.0	74.3	23.7
((PC,PY),(I3,(I1,(AJ2,(I2,AJ1)))));	0.156	138.9	17.7	87.6	25.0
((PC,PY),((I1,I3),(AJ1,(I2,AJ2))));	0.118	129.9	26.3	130.6	16.6
((PC,PY),(I1,(I3,(AJ1,(I2,AJ2)))));	0.081	130.5	47.3	130.5	47.3
((PC,PY),((I1,I3),(AJ2,(I2,AJ1))));	0.075	128.6	26.7	131.2	16.7
((PC,PY),(AJ1,((I2,AJ2),(I1,I3))));	0.046	65.1	13.4	88.2	11.3
((PC,PY),(I1,(I3,(AJ2,(I2,AJ1)))));	0.036	130.9	47.3	130.9	47.3
((PC,PY),(AJ1,(I2,(AJ2,(I1,I3)))));	0.034	57.4	11.8	81.1	10.0
((PC,PY),(I3,((I2,AJ2),(I1,AJ1))));	0.017	145.5	19.0	32.6	12.2
((PC,PY),(I3,(AJ1,(I1,(AJ2,I3)))));	0.014	145.5	20.1	32.6	17.0

Topology key	
PC	Puma_concolor_KP202261
PY	Puma_yagouaroundi_NC_028311
I3	Ind3_HyderabadZoo
I2	Ind2_Mysore
I1	Ind1
AJ1	Acinonyx_jubatus_NC_005212
AJ2	Acinonyx_jubatus_KP202271

Supplementary Table S4. Multiplex PCR primers used for amplicon sequencing.

Sequence Range	Primer ID	1st-PCRP	2nd-PCRP	Product Length
175- 275	rs201	ACGTTGGATGTGTACGCGTATACGTGGGTG	ACGTTGGATGACAGTCAAGGTGCTATTCAG	101
675-740	rs701	ACGTTGGATGGGCCTTATGTTTAGGTACGG	ACGTTGGATGCGTGTCTATTTATGTCCTGC	66
757- 859	rs701	ACGTTGGATGGGCCTTATGTTTAGGTACGG	ACGTTGGATGTACCAATCCCCTATCATCGC	103
1152- 1233	rs1101	ACGTTGGATGGGTCCTAGCCTTTCCATTAG	ACGTTGGATGTTAATCGTATGACCGCGGTG	82
1672- 1750	rs1501	ACGTTGGATGACTGCACGATAGCTAAGACC	ACGTTGGATGTTCTGGGTGTAAGCCAGATG	79
2851- 2923	rs2701	ACGTTGGATGCATGCCTGTGTTGGATTAAC	ACGTTGGATGTAATTGACCCAAAGAGACCC	73
3070- 3150	rs2901	ACGTTGGATGGATTATGCTACCTTTGCACG	ACGTTGGATGGATGTCCTGATCCAACATCG	81
3260- 3335	rs3101	ACGTTGGATGCCCCAACCTAAATTGTTGGC	ACGTTGGATGAAGGCCCACTTCATCAAAGC	76
4460- 4540	rs4301	ACGTTGGATGTGGCGTATTCTGCTAGAAAG	ACGTTGGATGGGTGTAGTAATGGTAGCACG	81
4640- 4730	rs4501	ACGTTGGATGAAAGCTAGGGTAAGAGGGAG	ACGTTGGATGTAGGAATGATGGCTAGTAGG	91
4865- 4930	rs4701	ACGTTGGATGACATAGAGGTTTAAATCCCC	ACGTTGGATGGGTACTCAGAAGTGAAAGGG	66
5481- 5536	rs5301	ACGTTGGATGCGTAGTTGTGTTTGGTTGAG	ACGTTGGATGGTTTGAGGGAAATATGGTTAG	56
5865- 5950	rs5701	ACGTTGGATGTAGCCATAACAGCACTACTC	ACGTTGGATGCTGGCTTCAATCCACTTCTC	86
6660- 6740	rs6501	ACGTTGGATGAAGAAAGAGGGAGGAAGGAG	ACGTTGGATGACTCCCAGTTTTAGCAGCAG	81
6910- 6990	rs6901	ACGTTGGATGTAAGATAGGATCTCCTCCTC	ACGTTGGATGTATGCTCGTGTGTCTACGTC	81
7248- 7330	rs7201	ACGTTGGATGATCAATTGGCTTCCTGGGCT	ACGTTGGATGGGGCAAAAATTCACTTCACG	83
7465- 7545	rs7301	ACGTTGGATGTCTAGTGAAGAGTTAGCCAG	ACGTTGGATGTTGCATCCAAGCGAGAAGTG	81
7670- 7695	rs7501	ACGTTGGATGTTCCAGATAGGCCTAGGAAG	ACGTTGGATGTTGCATCCAAGCGAGAAGTG	26
9240- 9330	rs9101	ACGTTGGATGAGCTCTAATACTGATGTCCC	ACGTTGGATGTGGGTTTGGTGGGTCATTAG	91
10268- 10375	rs10101	ACGTTGGATGTAGTCCGTGGAATCCTGTAG	ACGTTGGATGACGCAGAAAAAGTAAGCCCC	108
10680- 10781	rs10501	ACGTTGGATGTCATGGAAAAGGGCAAACGG	ACGTTGGATGGATTGTTCAGGACTACTATCG	102
11071- 11170	rs10901	ACGTTGGATGCCCACCTAATGTCTTCTCTC	ACGTTGGATGTTCTCAGACTCACTCTCTGC	100
11250- 11335	rs11101	ACGTTGGATGCCTCCTACAATGCTAAAAA	ACGTTGGATGATAGGAAATACAGGCCAGCG	86
12448- 12545	rs12301	ACGTTGGATGCTCGTTGGGTTGTAATGAGT	ACGTTGGATGTGCTAATTCATGCCTCCACG	98
12546- 12629	rs12501	ACGTTGGATGGTTCTTGCATACTTTTTCGG	ACGTTGGATGGTTGCCTTTATATAATCGGG	84
12641- 12720	rs12601	ACGTTGGATGTTTGGTTCCTAAGGCCAATG	ACGTTGGATGTGCTAATTCATGCCTCCACG	80

12769- 12847	rs12701	ACGTTGGATGGGAGACCTAGAATGTCCTTG	ACGTTGGATGGTAACAAATAGTGCTACGGG	79
12770- 12850	rs12701	ACGTTGGATGGGAGACCTAGAATGTCCTTG	ACGTTGGATGGTAACAAATAGTGCTACGGG	81
12870- 12940	rs12801	ACGTTGGATGGAGATGACTGTTTCCTGTCC	ACGTTGGATGGATATATACACTCAGATCC	71
12972- 13074	rs12901	ACGTTGGATGCCAAACCCTTAATCTATCAC	ACGTTGGATGAAATTGGTAAGGAACCATGC	103
13054- 13142	rs13001	ACGTTGGATGGGTTGTTGGCGGTTACTAGG	ACGTTGGATGAAATTGGTAAGGAACCATGC	89
13353- 13445	rs13301	ACGTTGGATGCCCAACATGACAACCTAAAC	ACGTTGGATGTACAAACCCTCACCCTATGC	93
13475- 13552	rs13401	ACGTTGGATGCTACCATCAGCCATAGAAGG	ACGTTGGATGCTTCTCAACCTCAAGCCAAC	78
14031- 14123	rs14001	ACGTTGGATGTATCCTGCAAAGATACTTCC	ACGTTGGATGCGATTCAACGCCTTGAATCC	93
14510- 14530	rs14501	ACGTTGGATGAATCACCCTAACCCTTAGCC	ACGTTGGATGCTCCTCACTAAAAAACCCCG	21
15253- 15327	rs15101	ACGTTGGATGGAGCCGAAGTTTCATCATGC	ACGTTGGATGGCCGATGTAAGGAATTGCTG	75
15670- 15760	rs15501	ACGTTGGATGGGATATGTCTTACCATGAGG	ACGTTGGATGTCCTCCCCATATTAAACCCG	91
15851- 15945	rs15701	ACGTTGGATGGGAGACCTAGAATGTCCTTG	ACGTTGGATGCACCCTAACATGAATTGGCG	95
16061- 16130	rs15901	ACGTTGGATGTATTAGGGCTAGGACTCCTC	ACGTTGGATGGTGAGTTCTCCTTTTTTGGC	70
16271- 16354	rs16201	ACGTTGGATGTCAGGCATCATCGAAAACCG	ACGTTGGATGCCCTGCTAATACCAGAAACC	84
16447- 16538	rs16401	ACGTTGGATGTGTTGGAAGTGAGGACATAC	ACGTTGGATGCCCTGCTAATACCAGAAACC	92

References

Burger, P. A., Steinborn, R., Walzer, C., Petit, T., Mueller, M., and Schwarzenberger, F. (2004). Analysis of the mitochondrial genome of cheetahs (*Acinonyx jubatus*) with neurodegenerative disease. Gene, **338(1):111-119**.

Charruau, P., Fernandes, C., Orozco-Terwengel, P., Peters, J., Hunter, L., Ziaie, H., Jourabchian, A., Jowkar, H., Schaller, G., Ostrowski, S., *et al*. (2011). Phylogeography, genetic structure and population divergence time of cheetahs in Africa and Asia: evidence for long-term geographic isolates. Molecular Ecology, **20(4):706-724**.

Freeman, A. R., Machugh, D. E., McKeown, S., Walzer, C., McConnell, D. J., and Bradley, D. G. (2001). Sequence variation in the mitochondrial DNA control region of wild African cheetahs (*Acinonyx jubatus*). Heredity, **86(3):355**.

Li, G., Davis, B. W., Eizirik, E., and Murphy, W. J. (2016). Phylogenomic evidence for ancient hybridization in the genomes of living cats (*Felidae*). Genome Research, **26(1):1-11**.
